# Impaired Ciliogenesis in differentiating human bronchial epithelia exposed to non-Cytotoxic doses of multi-walled carbon Nanotubes

**DOI:** 10.1186/s12989-017-0225-1

**Published:** 2017-11-13

**Authors:** Ryan J. Snyder, Salik Hussain, Charles J. Tucker, Scott H. Randell, Stavros Garantziotis

**Affiliations:** 1 National Institutes of Health (NIH), National Institute of Environmental Health Sciences (NIEHS), Research Triangle Park, Durham, NC 27709 USA; 2 University of North Carolina Chapel Hill, Chapel Hill, NC 27599-7248 USA

**Keywords:** Carbon Nanotubes, Cilia, Ciliogenesis, Epithelial cells

## Abstract

**Background:**

Multi-walled carbon nanotubes (MWCNTs) are engineered nanomaterials used for a variety of industrial and consumer products. Their high tensile strength, hydrophobicity, and semi-conductive properties have enabled many novel applications, increasing the possibility of accidental nanotube inhalation by either consumers or factory workers. While MWCNT inhalation has been previously shown to cause inflammation and pulmonary fibrosis at high doses, the susceptibility of differentiating bronchial epithelia to MWCNT exposure remains unexplored. In this study, we investigate the effect of MWCNT exposure on cilia development in a differentiating air-liquid interface (ALI) model. Primary bronchial epithelial cells (BECs) were isolated from human donors via bronchoscopy and treated with non-cytotoxic doses of MWCNTs in submerged culture for 24 h. Cultures were then allowed to differentiate in ALI for 28 days in the absence of further MWCNT exposure. At 28 days, mucociliary differentiation endpoints were assessed, including whole-mount immunofluorescent staining, histological, immunohistochemical and ultrastructural analysis, gene expression, and cilia beating analysis.

**Results:**

We found a reduction in the prevalence and beating of ciliated cells in MWCNT-treated cultures, which appeared to be caused by a disruption of cellular microtubules and cytoskeleton during ciliogenesis and basal body docking. Expression of gene markers of mucociliary differentiation, such as FOXJ1 and MUC5AC/B, were not affected by treatment. Colocalization of basal body marker CEP164 with γ-tubulin during days 1–3 of ciliogenesis, as well as abundance of basal bodies up to day 14, were attenuated by treatment with MWCNTs.

**Conclusions:**

Our results suggest that a single exposure of bronchial cells to MWCNT during a vulnerable period before differentiation may impair their ability to develop into fully functional ciliated cells.

**Electronic supplementary material:**

The online version of this article (10.1186/s12989-017-0225-1) contains supplementary material, which is available to authorized users.

## Background

Nanomaterial science has made significant advancements over the past decade, taking advantage of the unique properties of nanoscale substances to enable a staggering variety of novel applications and products. Multi-walled carbon nanotubes (MWCNTs) are among the most commonly used engineered nanomaterials, and their commercial production has increased significantly in recent years [1]. MWCNTs are nanoscale overlapping cylinders of graphene, manufactured for their high tensile strength and hydrophobic properties. These properties have made them especially useful in industrial applications and consumer products such as reinforced concrete [2], sporting equipment [3], textiles [4], spray-on coatings [5], and other uses [6].

However, the stiff and fiber-like nature of MWCNTs has raised concerns about their safety. Similarities to asbestos fibers [7], as well as cytotoxic and pro-inflammatory effects [8] have been demonstrated in a number of studies. MWCNTs enter epithelial cells and have been found in the cytoplasm [9], mitochondria [10], and endosomes [11]. Inhalation of MWCNTs induces pulmonary inflammation and fibrosis [12], and retained fibers have been found in the lungs of exposed rodent models months after MWCNT exposure [13]. These adverse health effects were demonstrated at high doses; however, even lower, more occupationally-relevant doses can have significant effects on pulmonary cells [14, 15]. We have previously demonstrated effects of MWCNTs on the barrier function of primary human BECs and cytoskeletal disruptions resulting from non-cytotoxic exposures [16] in submerged cultures. Impaired barrier function in the pulmonary epithelium of MWCNT-exposed individuals may further increase their susceptibility to environmental insult. These effects may be particularly important in susceptible individuals with pre-existing lung disease, or in cases of long-term chronic exposure. Denuded epithelium resulting from tobacco smoke [17] or viral infection [18] could expose undifferentiated basal cells to nanoparticles, potentiating further injury or attenuating epithelial regeneration.

MWCNT-induced effects on the cytoskeleton are of particular interest in the context of pulmonary exposure, because of the importance of actin and tubulin networks in the organization, orientation, docking, and structure of airway cilia. Multiciliated cells differentiate from epithelial precursors in the conducting airways and are critical to the removal of debris and pathogens from the lung [19]. While an intact, healthy, functioning mucociliary epithelium constitutes a significant barrier to inhaled particulates [20], exposed epithelial precursors in an injured lung may be more susceptible to MWCNT effects [21]. Carbon nanotubes can damage the cytoskeletal network [22] or have steric interactions [23] with microtubules and may therefore have detrimental effects on cilia formation and/or function even after exposure has ceased.

This study investigated whether undifferentiated cell exposure to MWCNT affects the cells’ differentiation potential. We utilized a brief, 24-h exposure to non-cytotoxic doses of MWCNTs during submerged, undifferentiated culture and evaluated subsequent mucociliary differentiation. Primary BECs from healthy human donors were exposed to MWCNT in confluent submerged culture, then converted to an air-liquid interface (ALI) model and allowed to differentiate for 4 weeks in the absence of further MWCNT exposure. Mucociliary differentiation in MWCNT-treated cultures was compared to dispersion vehicle-treated and graphitized carbon-treated controls. We found that a single 24-h exposure of undifferentiated cells to MWCNTs decreased formation and function of cilia in differentiated cells analyzed 28 days later. To our knowledge, this is the first demonstration that MWCNT exposure during the vulnerable undifferentiated state may perturb cell development, and suggests that windows of susceptibility may exist for specific MWCNT effects.

## Results

### Attenuated cilia staining in differentiated ALI culture 28 days after single MWCNT exposure

**Fig. 1:**
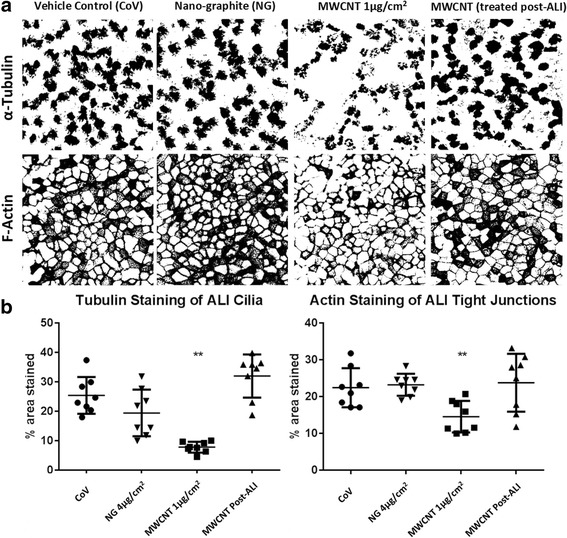
**a** Binary images of day 28 mucociliary BECs treated with control vehicle (CoV), mesoporous graphitized nanocarbon (NG) or multi-walled carbon nanotubes (MWCNTs) for 24-h. Cells were treated while undifferentiated, and allowed to differentiate in air-liquid interface for 28 days after ending treatment. A “post-ALI” MWCNT 1μg/cm^2^ treatment is included for comparison, in which treatment was delayed until after the BECs had differentiated for 20 days in ALI. Tubulin staining in cilia (top) and F-actin staining in tight junctions (bottom) demonstrate reduction of differentiated cells resulting from MWCNT treatment. These images are examples of those used for pixel density analysis, representing a summation image from the top 10 μm of epithelium. Intensity below a threshold value was excluded from the binary image. **b** Results of image quantification of randomized sections of membrane. There is a significant (*p < 0.05, **p < 0.01 ANOVA, Bonferroni post hoc) reduction in α-tubulin and F-actin translocation to cilia and tight junctions (respectively) following MWCNT treatment. Data compiled from 3 donors, with 2–3 replicate wells per donor. Bars represent mean ± standard deviation (SD)

We first analyzed the effect of a single MWCNT exposure on cell differentiation. Characterization of the particles used in this study can be found in the Methods and in Additional file 1. We did not find any cytotoxicity from our MWCNT at any used dose, based on LDH release (Additional file 2). Ciliated cell area in 28-day differentiated ALI cultures was attenuated in MWCNT-treated BECs compared to vehicle controls (Fig. 1). The area covered by fluorescently-labeled ciliary α-tubulin was reduced from 25.46 ± 6.25% (Mean ± SD) in control cultures to 7.83 ± 1.84% in the MWCNT-treated cultures (*p* < 0.0001). Analysis of F-actin staining coverage also revealed a significant decrease in tight junction actin from 22.43 ± 4.56% to 14.36 ± 3.93% (*p* < 0.006). By contrast, cells which were treated with MWCNTs after they had differentiated in ALI for 21 days had ciliary α-tubulin and tight junction actin staining of 32.07 ± 7.32% and 23.83 ± 7.86% (respectively), and were not significantly different from control cultures.

### Reduced prevalence of ciliated cells compared to goblet cells in differentiated air-liquid interface culture 28 days after MWCNT exposure

**Fig. 2:**
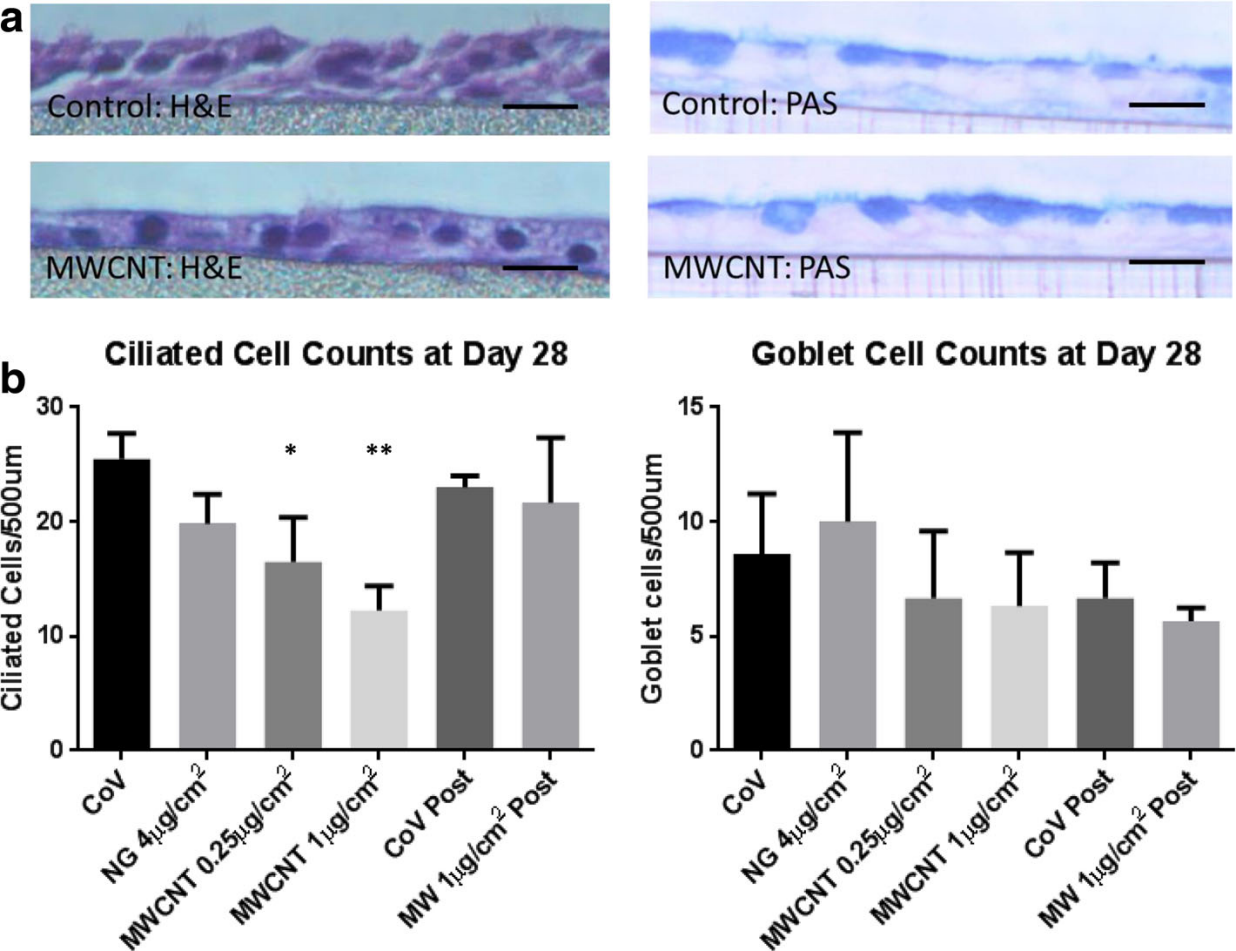
**a** Histological cross-sections of differentiated cultures, stained with H&E (left) and PAS (right) for counting ciliated and goblet cells respectively. Representative images from vehicle controls (top) and MWCNT 1 μg/cm^2^ (bottom) are shown here. Scale bars represent 10 μm. **b** Ciliated and goblet cell counts of stained cultures. Ciliated and goblet cells per 500 μm length of membrane were counted for each treatment and counts from all 3 donors were pooled. Vehicle control (CoV) and MWCNT 1 μg/cm^2^ treatments performed post-differentiation (at day 20) were included for comparison (labeled “Post”). A significant (*p < 0.05, **p < 0.01 ANOVA, Bonferroni post hoc) dose-dependent decrease in ciliated cells was observed by day 28, though this decrease was not seen in goblet cells. Data compiled from 3 donors, with 5 slide sections per donor. Bars represent mean ± standard deviation (SD)

To determine whether MWCNT exposure specifically affected the formation of ciliated cells or could affect multiple cell lineages, we also studied the differentiation of mucin-secreting goblet cells. Cross-sections of paraffin-embedded membranes were used to count both ciliated and goblet cells. Sections from differentiated 28-day cultures revealed a dose-dependent decrease in ciliated cell counts with increasing MWCNT dose (Fig. 2). Pooled counts from NG-treated cells had an average of 27.75 ± 4.93 (mean ± SD) ciliated cells per 500 μm section, while the sections from cultures treated with MWCNT 1 μg/cm^2^ contained an average of 13.65 ± 3.26 ciliated cells per 500 μm (*p* < 0.003). Goblet cells, by contrast, were not significantly affected by MWCNT exposure and were observed in equivalent abundance between control (8.6 ± 2.62 goblet cells per 500 μm section) and 1 μg/cm^2^ MWCNT-treated (6.3 ± 2.31) ALI cultures.

### MWCNT treatment induced heterogeneity in cilia beating frequency

**Fig. 3:**
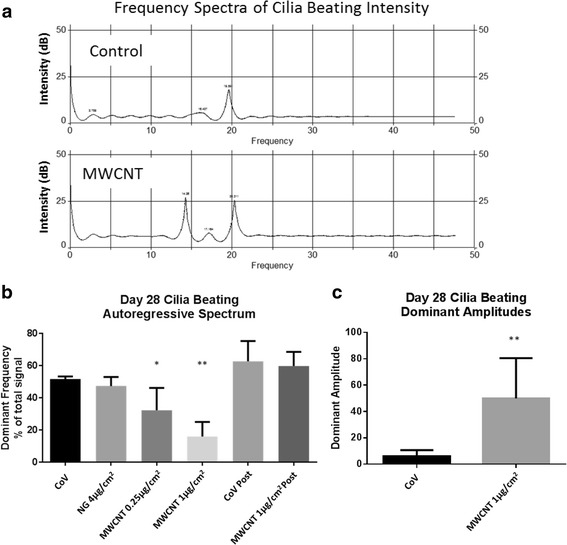
**a** Autoregressive spectra of cilia beating frequency in control (CoV) vs MWCNT 1 μg/cm^2^, showing representative examples of intensity peaks. Dominant frequencies were typically close to 20 Hz and these peaks contained most of the signal, however, in MWCNT-treated cells, a larger percentage of the total signal was found at other frequencies. **b** Percentage of the total signal which fell under the dominant beating frequency peak decreased with MWCNT exposure in a dose-dependent manner. Significantly more of the total beating frequencies were found outside of the dominant 20 Hz signal in MWCNT-exposed BECs, indicating greater heterogeneity of cilia beating. Vehicle control (CoV) and MWCNT 1 μg/cm^2^ treatments performed post-differentiation (at day 20) were included for comparison (labeled “Post”). **c** Dominant amplitude of the cilia beating pattern also changed with MWCNT exposure, with greater variability in amplitude found in MWCNT-exposed cells. (*p < 0.05, **p < 0.01 ANOVA, Bonferroni post hoc, bars indicate SD). Data for intensity and amplitude measurements were compiled from 2 donors each, with 5 motion-captures from each donor

Having shown that MWCNT exposure affects cilia development, we wanted to investigate MWCNT effects on cilia function. We therefore analyzed the effect of MWCNT exposure on cilia beating by measuring the frequency of intensity changes in motion capture video taken at the center of each insert. Control inserts developed cilia with a relatively uniform beating frequency and produced a dominant peak at approximately 20 Hz accounting for an average of 51.6 ± 1.76% (mean ± SD) of the total signal (Fig. 3). Cultures which had been pretreated with MWCNT also had a dominant peak at 20 Hz; however, the percentage of the total cilia beating signal which fell beneath this dominant peak was decreased in a dose-dependent manner. Ciliated cells that formed in the inserts previously treated with 1 μg/cm^2^ MWCNTs possessed a much wider range of beating frequencies, with the dominant 20 Hz peak accounting for only 16.07 ± 9.07% of the total signal. Additionally, the amplitude of cilia beating intensity was greatly elevated in MWCNT-treated cells (Fig. 3c), at 45.7 ± 30.1 dB (standard deviation of peak amplitude) compared to 5.14 ± 3.97 dB in control wells.

### Gene expression in ALI cultures is unaltered by early MWCNT exposure

**Fig. 4:**
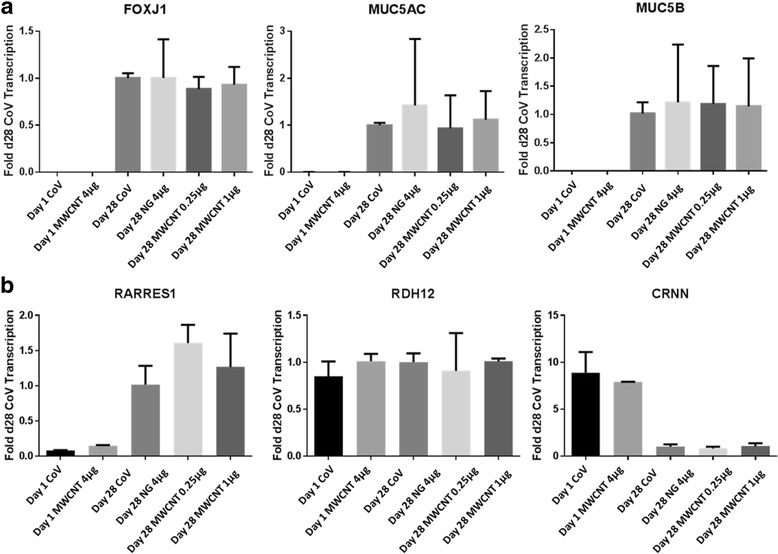
**a** Expression of ciliogenesis marker gene *FOXJ1* and mucin genes *MUC5AC* and *MUC5B* in ALI day 1 (“d1”) and ALI day 28 (“d28”) BECs, following a 24 h exposure to MWCNTs during submerged culture. Bars indicate standard deviation and treatments are expressed as μg/cm^2^. Day 1 expression of these markers was over 1000-fold less than at day 28 in control and MWCNT treatments. By day 28, no treatment-related changes in *FOXJ1* expression were observed in cells from any of the 3 donors. **b** Expression of other genes related to mucociliary differentiation signaling was also unaffected by MWCNT treatment, both at ALI day 1 and day 28 following exposure. Retinoid signaling genes *RARRES1* and *RDH12* were unaltered by MWCNTs or NG treatments. Cornulin (*CRNN*) was downregulated after 28 days in ALI culture in all treatment groups. Data was compiled from 3 donors, with significance determined by ANOVA (*p*-value >0.05 following Bonferroni comparison)

We then investigated the mechanism of MWCNT inhibition of cilia development and function. We analyzed expression of genes that are known to be associated with differentiation of ciliary or goblet cells. *FOXJ1*, *MUC5AC* and *MUC5B* genes were analyzed in ALI converted cultures 1 day after MWCNT exposure, as well as in ALI day 28 cultures (Fig. 4). *FOXJ1*, *MUC5AC* and *MUC5B* expression was not affected by any of the MWCNT treatments at any time point, suggesting that the attenuation of cilia by MWCNTs was not mediated via changes in gene expression. We also examined the expression of retinoid signaling marker genes *RARRES1* and *RDH12*, as well as keratinocyte marker *CRNN*, and found no MWCNT-induced changes in these genes at either day 1 or day 28 following exposure.

### MWCNT is associated with modest ciliary axoneme abnormalities

**Fig. 5:**
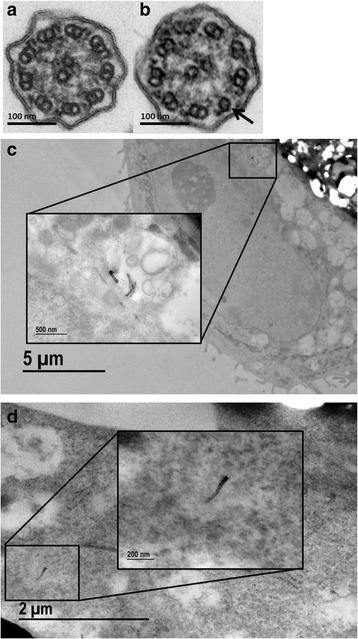
Abnormal microtubule arrangements in cilia cross-sections. Ciliary axoneme abnormalities were tallied in 224 control-treated and 234 MWCNT-treated cross-sections from a single donor. Sections were categorized as normal cilia (represented in panel **a**), axoneme abnormalities (represented in panel **b**, arrow points to singlet), or dynein arm abnormalities (not found). While MWCNT treatment increased axoneme abnormalities above control levels by 3-fold (6.0% vs 1.9%), the absolute numbers of abnormal cilia in MWCNT treated cells are not sufficient to alter cilia beating/clearance. Ultrastructural imaging was also used to verify the presence of MWCNTs within the cell 24 h after treatment of undifferentiated cultures. MWCNT fibers are observed in vesicular structures (**c**) and free in the cytoplasm (**d**)

Elongation of motile cilia depends on an intact microtubule axoneme structure and unobstructed dynein arm movement [24]. While previous research has effectively demonstrated physical interaction and biomimicry [23] between carbon nanotubes and intracellular microtubules, interactions with ciliary microtubules has not yet been shown. To investigate whether MWCNT exposure alters axoneme structure and, by this mechanism, impairs development of normal cilia, cross sections of cilia from day 28 BECs treated with MWCNT 1 μg/cm^2^ were compared to those from control cells by TEM (Fig. 5). Axoneme abnormalities from the typical “9 + 2” arrangement appeared elevated with MWCNT treatment, occurring in 14 out of 234 cross sections examined (6.0%), compared to 4 out of 224 control cilia (1.9%). Dynein arm abnormalities were not observed in either treatment.

### Disruption of actin web formation and apical translocation of basal bodies by MWCNTs

**Fig. 6:**
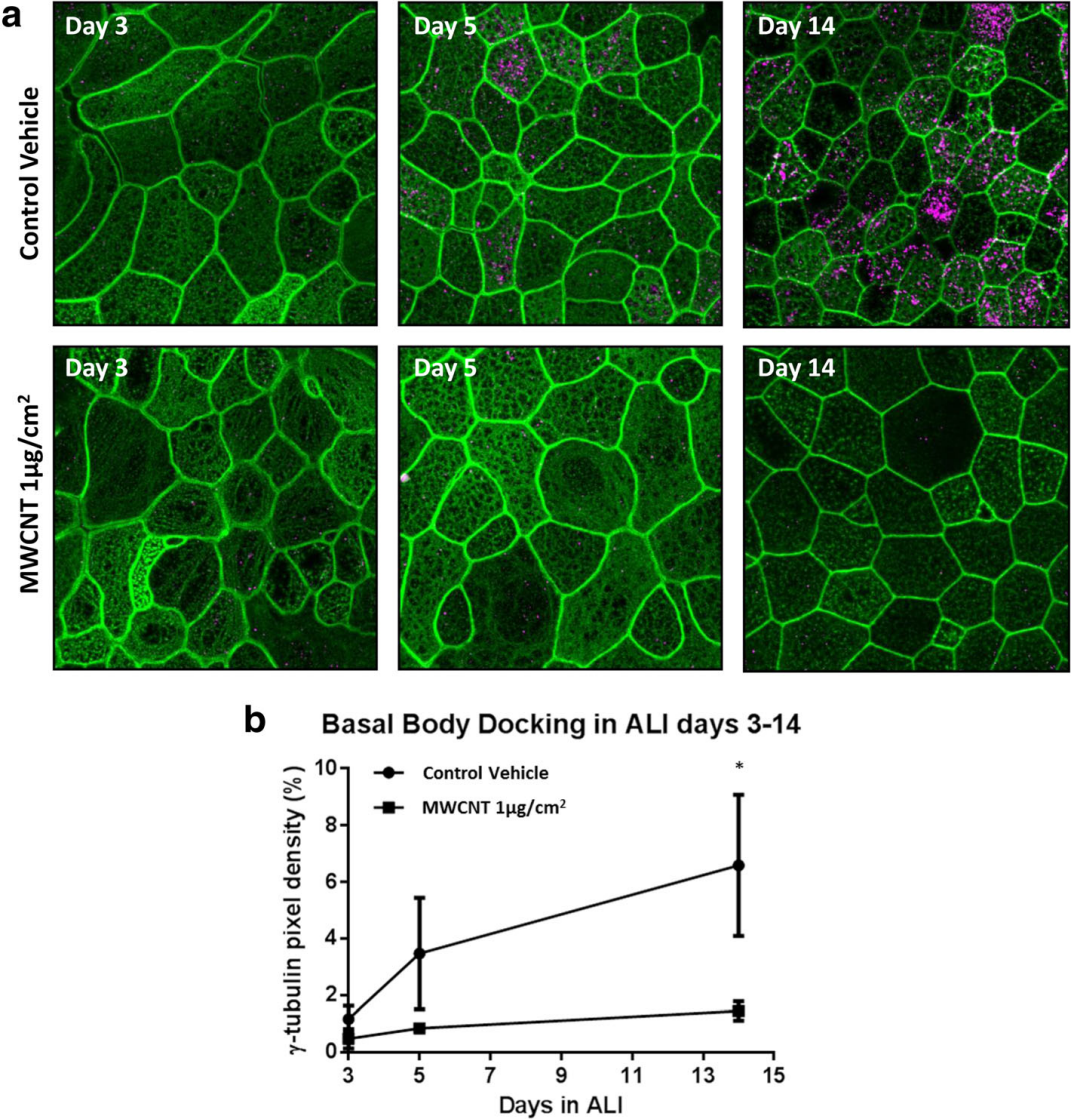
**a** Staining of F-actin (green) and γ-tubulin (magenta) in ALI cultures undergoing early ciliogenesis at days 3, 5 and 14. MWNCT-treated cultures exhibited disorganized actin structure at ALI day 3, prior to apical localization of γ-tubulin. By day 5, extensive staining of γ-tubulin in apical basal bodies was observed in control wells, while MWCNT-treated wells retain γ-tubulin staining primarily in centrioles. By ALI day 14, both MWCNT and vehicle-treated cultures are developing cilia, though basal body staining remains noticeably less pronounced in cells previously exposed to MWCNTs. **b** Quantitative pixel area analysis of γ-tubulin staining in combined images from 3 donors (bars represent standard error). Appearance of tubulin-staining basal bodies at the apical surface of the BECs by day 5 was significantly decreased by MWCNT exposure at day 1. (*p < 0.05, Multiple t-test with Holm-Sidak comparison correction)

We evaluated apical actin web morphology and appearance of γ-tubulin-staining basal bodies during early ciliogenesis to determine whether MWCNT exposure could affect cilia docking. Confocal images of actin cytoskeleton in ALI days 3, 5, and 14 show disorganized formation of the apical cytoskeletal web by day 3 in cultures treated with MWCNT 1 μg/cm^2^ compared to vehicle controls (Fig. 6). Basal bodies, stained via γ-tubulin (magenta), were apparent and frequent in the day 5 control cultures, but tubulin staining remained mostly in intracellular centrioles by the same time point in cultures treated with MWCNTs. By day 14, MWCNT-treated cultures had also developed basal bodies, but with greatly reduced occurrence compared to control cultures. Pixel area analysis (using Image J) of maximum intensity projections from these confocal images was used to quantify the docking of basal bodies (Fig. 6b). By day 14, basal body docking was significantly reduced in MWCNT-exposed BECs compared to control cultures (*p* < 0.05, Multiple t-tests, Holm-Sidak comparison correction).

### Attenuated colocalization of CEP164 and γ-tubulin in basal bodies during early ciliogenesis

**Fig. 7:**
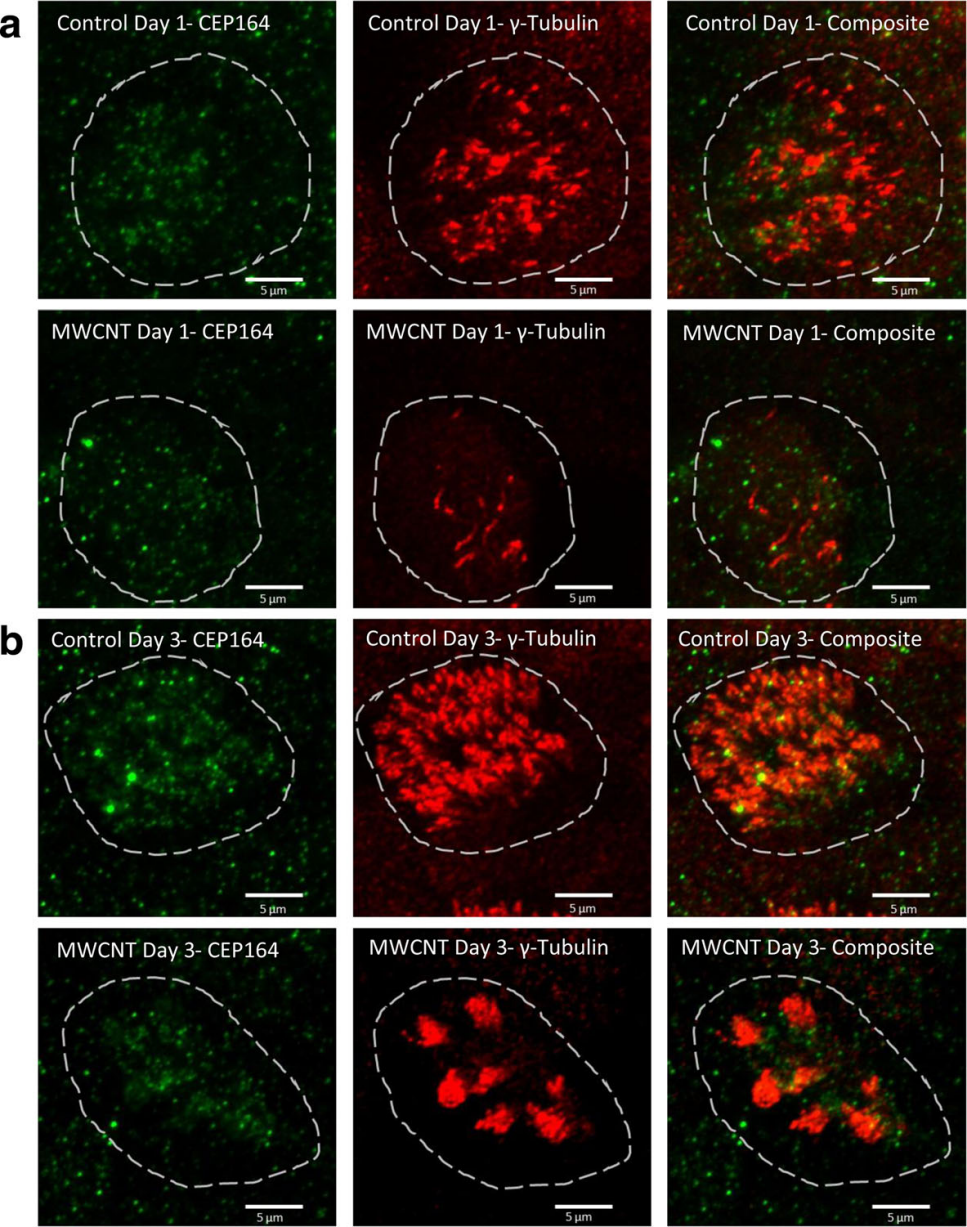
Colocalization of CEP164 and γ-tubulin during early ciliogenesis at days 1 (**a**) and 3 (**b**) following removal of apical medium. Basal body marker CEP164 colocalizes with γ-tubulin at the apical cell surface in control BEC cultures and forms a uniform spread of ciliary precursors across the cell. In MWCNT-treated cultures, CEP164 staining is more attenuated and patchy, resulting in fewer, more clumped cilia. Each image is centered on a single cell, with approximate borders demarcated by a dotted line. Scale bars indicate 5 μm

We examined the apical surfaces, to a depth of 6 μm, of individual cells at post-ALI days 1 and 3 for CEP164 staining and colocalization with γ-tubulin in the ciliary necklace/transition zone. In Fig. 7, we show that BECs treated with 1 μg/cm^2^ MWCNTs had noticeably less CEP164 staining, and that its colocalization with γ-tubulin was patchy and inconsistent compared to the vehicle-treated BECs. This effect was apparent with MWCNT treatment at post-ALI day 1 and by day 3 resulted in defined “clusters” of ciliary precursors on the apical surface despite having wider coverage of CEP164.

### Trans-epithelial electrical resistance (TEER) is unaffected by MWCNT exposure

**Fig. 8:**
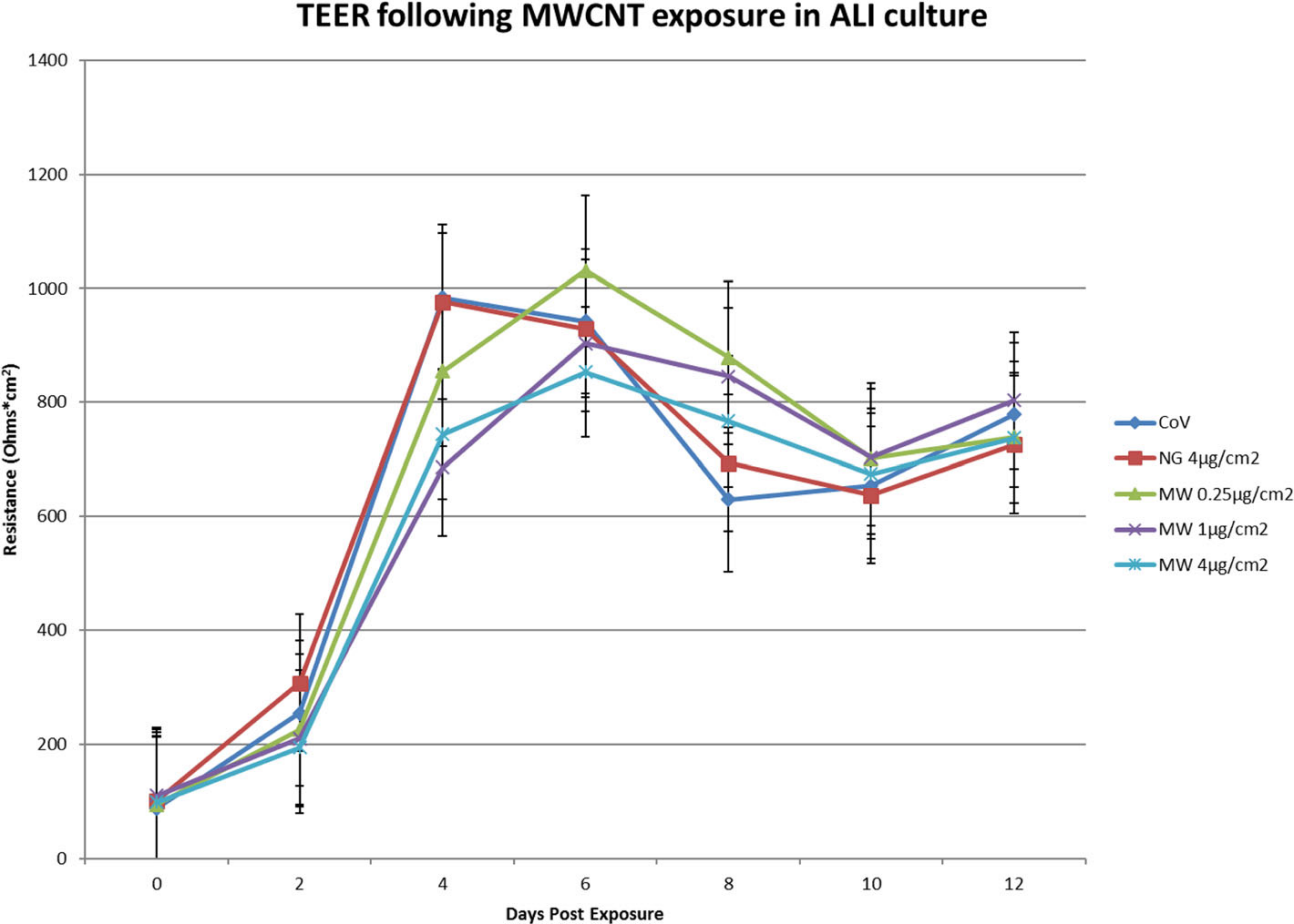
Trans-epithelial electrical resistance (TEER) from days 0–12 following treatment and removal of apical medium. Measurements are expressed as ohms*cm^2^ and were recorded every 2 days prior to feeding, starting with the treatment medium at day 0 immediately before transfer to ALI. All cultures, regardless of treatment, appeared to increase in TEER rapidly over the first week, then plateau between 700 and 900 ohms*cm^2^. No statistically significant differences (by ANOVA) in TEER progression between treatments were found at the doses used in this study, despite the observed effects of MWCNTs on ciliary differentiation. Data compiled from 3 replicate wells from a single donor. Bars represent mean ± standard deviation (SD)

As the actin translocation to cell junctions appeared attenuated by MWCNT exposure, we measured TEER in exposed ALI cultures to determine whether the barrier function of the differentiated cells could also be impaired. In Fig. 8, TEER measured in ALI cultures treated pre-differentiation with MWCNTs or controls is recorded every 2 days after treatment until day 12, averaging the measurements from 3 donors. TEER from all ALI cultures increases rapidly during differentiation. However, no significant effect from any treatment was observed on the TEER of differentiating cultures, suggesting that epithelial barrier function is retained at the study doses despite the MWCNTs’ effect on developing cilia. Staining for ZO-1, a tight junction-specific marker, showed no significant treatment effect on junction staining, supporting the TEER results (Additional file 3).

## Discussion

Taken together, our results suggest that a brief, 24-h exposure of undifferentiated BECs to low, non-cytotoxic doses of MWCNTs is sufficient to specifically impair ciliogenesis during air-liquid interface differentiation. The abundance of ciliated cells in exposed cultures is attenuated and cilia beating frequencies remain heterogeneous and disrupted at 28 days following the end of MWCNT exposure. This implies that exposure to MWCNTs during a vulnerable period can permanently affect differentiation of precursor epithelial cells in the conducting airways.

Whole-mount staining reveals that total cilia coverage in differentiating BECs is reduced by a brief 24-h MWCNT exposure during submerged culture 28 days prior. As the MWCNTs were no longer present in the culture medium by the time the cultures were converted to ALI, intracellular changes induced by the MWCNT exposure influenced later ciliogenesis and differentiation. Additionally, F-actin staining was also reduced in the MWCNT-treated cultures. While this did not have an impact on the tight junctions or barrier function (see Fig. 8 and Additional file 3), altered intracellular distribution of F-actin is consistent with our previous research [16] in submerged cultures, and also found by other researchers [25] investigating carbon nanotubes. Interactions between carbon nanotubes and cytoskeletal components have been described previously, both for actin fibers [26] and microtubules [23, 27]. Our results suggest that cytoskeletal involvement in ciliogenesis may be particularly susceptible to sustained effects of MWCNT exposure.

In addition to a reduction in cilia, BECs which differentiated after MWCNT treatment had altered cilia beating behavior. A significant decrease in the percentage of normal cilia beating frequencies was observed in treated cultures, as well as a significant increase in amplitude. These results indicate a loss of uniformity in ciliary beating in cells which developed cilia after MWCNT exposure, compared to controls. The concerted motion of beating cilia is critical to efficient clearance of mucus by the mucociliary escalator in vivo [28]. Therefore, this finding could have implications on the buildup of mucus and particulates in the MWCNT-exposed lung.

Counts of ciliated and goblet cells in PAS and H&E-stained cross sections also reveal that the effect of MWCNT treatment appears to be confined to ciliated cells alone. The finding that goblet cells are not affected suggests that general differentiation signaling in BECs is not impaired by MWCNTs, and the effect is specific to ciliogenesis. This is further supported by the lack of any MWCNT-induced effects on the expression of various differentiation gene markers, such as *FOXJ1*, *MUC5AC/B*, *RDH12*, and *RARRES1*. These results, combined with negative results from a previous microarray analysis (not shown), suggest that the mechanism by which MWCNTs impair ciliogenesis is not transcriptionally mediated and is independent of differentiation signaling cascades. We therefore investigated interactions between MWCNTs and the synthesis and docking of basal bodies during early ciliogenesis.

Ultrastructural imaging of the cilia which developed in treated and control BECs revealed a modest increase in axoneme abnormalities, from 1.9% of counted cross sections in controls to 6.0% after MWCNT treatment. This suggests that MWCNTs may interfere with ciliary axoneme formation, which could contribute to the observed ciliary dysfunction. However, normal ciliary function is thought to be retained when there are less than 10% abnormal/dysfunctional cilia [29]. Therefore, we investigated other effects of MWCNT exposure on early cilia formation.

The docking of basal bodies with the apical cell membrane is necessary for the development of motile cilia [30] and depends on interaction between the actin cytoskeletal web and basal body precursor components (procentriole and ciliary vesicle). Disruption of the cytoskeletal web by carbon nanotubes has been described previously [26], and our findings in Fig. 6 confirm that a similar effect can be replicated in our cell cultures using the multi-walled nanotubes implemented in this study. As cytoskeletal disruption was observed prior to the appearance of γ-tubulin-staining basal bodies in treated cultures, MWCNTs may be disrupting early ciliogenesis events leading to docking. Further, as γ-tubulin staining of ciliary basal bodies is attenuated in MWCNT-exposed cells as early as ALI day 5, at least a week prior to the appearance of mature cilia in culture, our results support that the intracellular mechanism mediating impaired ciliogenesis occurs during or prior to basal body docking.

During very early ciliogenesis, CEP164 bound to procentrioles forms basal bodies and assists their docking to the apical membrane [31]. This allows CEP164 to be used as a biomarker for basal bodies prior to the development of the γ-tubulin-rich transition zone. In this study, we found that γ-tubulin staining and CEP164 staining at the apical surface of treated cells were poorly colocalized, defining a more specific molecular target for MWCNT-induced disruption of basal body synthesis in multiciliated cells. While in control BECs most CEP164 staining is colocalized with γ-tubulin and permits the formation of cilia, much of the CEP164 staining found in MWCNT-treated BECs is not associated with γ-tubulin at the transition zone. Therefore, it appears that MWCNTs may be interfering with the development of the ciliary transition zone during basal body synthesis. In combination with the disruption of the apical cytoskeleton, these molecular events could have a significant impact on early ciliogenesis given a brief exposure to MWCNTs.

In contrast to the nanotubes, mesoporous graphitized nano-scale carbon (NG) induced no significant effects on cell differentiation or ciliary function. The NG controls were applied to BECs at four-fold higher concentration than the highest MWCNT treatments, but ciliated cell abundance and beating frequencies remained unchanged from vehicle control values. This result strongly suggests that the mechanism for MWCNT-driven impairment of ciliogenesis is dependent on the unique tube structure and/or aspect ratio of the nanotubes. Biomimicry between carbon nanotubes and microtubules, on account of their similar shape and size, has been reported previously [15, 23] and physical interactions between nanotubes and cytoskeletal components remain a subject of active research [25, 32].

Our cilia-specific findings contrast with those of Boublil et al. [33] who found mucociliary differentiation, as a whole, to be modulated by ultrafine particulate exposure. We believe that our methodology differs from theirs in several important ways, which may account for the discrepancy. The materials used in the Boublil study (while nano-scale and carbon-based) were taken from exhaust and ambient particulates and contain residual organic compounds. These particles also lack the tubular shape and high aspect ratio of the carbon nanotubes used for this study, and would therefore not be expected to physically interact with cytoskeletal components as nanotubes are known to do. Finally, while our study dosage was restricted to a single 1 μg/cm^2^ exposure prior to conversion to air-liquid interface, the Boublil study applied doses up to 10 μg/cm^2^ every week until differentiation. Consequently, we do not believe there is any conflict or overlap between these studies or their endpoints.

## Conclusions

The results of our study are consistent with an attenuation of ciliogenesis and ciliary beating in BECs previously exposed to non-cytotoxic doses of MWCNTs during their undifferentiated phase. Since we do not find prospective loss of cilia in fully-differentiated cells which were exposed to MWCNTs, our results suggest that undifferentiated cells are especially vulnerable to MWCNTs, in particular with regard to ciliogenesis. Undifferentiated basal cells can become exposed to exogenous substances following airway injury, when the differentiated columnar cells are denuded [34]. Our findings therefore suggest that pre-existing lung injury (e.g. by noxious or infectious agents) could compound the adverse effects of MWCNT at concentrations which are not normally harmful; furthermore, chronic, low-dose MWCNT exposure may have long-lasting effects on ciliary differentiation and mucociliary clearance. Additional studies to investigate this previously-unreported mechanism of carbon nanotube-induced lung injury are needed to assess their long-term impact on ciliary development and lung health in vivo.

## Methods

### Nanomaterial characterization

Characterization of the multi-walled carbon nanotubes (MWCNTs) used for this study has been previously published [16, 35]. In brief, MWCNTs were purchased from Helix Nanomaterial Solutions and synthesized by chemical vapor deposition. Elemental carbon content was >95%, with catalytic metal impurities of nickel and lanthanum at <0.2 and <0.1% respectively. Nanotubes measured 10-30 nm in diameter and 500-4000 nm in length, as determined by TEM.

Mesoporous graphitized nanocarbon (NG) was used at 4 μg/cm2 as a control particle, as it possesses a similar graphitized surface chemistry without the tubular shape and aspect ratio of MWCNTs. NG was purchased from Sigma Aldrich (St. Louis, MO) and was of high purity (>99%).

Further information on the characterization of the nanomaterials used in this study can be found in Additional file 1.

### Cell culture and treatment

Primary human bronchial epithelial cells (BECs) were taken from bronchoscopy brushings of healthy donors (Additional file 1). Cells were grown in submerged culture using BEGM medium (Lonza) and frozen after 1 passage. Thawed BECs were seeded onto 12 mm Millicell standing porous inserts (EMD Millipore, Darmstadt, Germany) at ~10^5^ cells/cm^2^ in ALI differentiation medium [36] (University of North Carolina, Cystic Fibrosis Laboratory). Human collagen IV was used to coat insert membranes 24 h prior to seeding. Cultures were allowed to reach confluence while submerged in ALI medium prior to treatment with nanomaterials. Cultures were then treated in the apical chamber only with 0, 0.25, or 1 μg/cm^2^ (0, 0.75, and 3 μg/ml respectively) MWCNTs or 4 μg/cm^2^ (12 μg/ml) NG dispersed in ALI medium supplemented with 10 μg/ml 1,2-dipalmitoyl-sn-glycero-3-phosphocholine (DPPC) and 600 μg/ml sterile bovine serum albumin (BSA). Both nanomaterial suspensions and control media were dispersed via sonication in a cup horn (Misonix, Farmingdale, NY) at amplitude 100 in 5 pulses of 3 min each, replacing the cup horn with cold water between pulses. Dynamic light scattering (DLS) measurements of nanotube agglomerates in this vehicle solution had hydrodynamic diameters between 10 and 200 nm, and graphitized carbon agglomerates formed between 20 and 150 nm, as previously shown [16]. Nanomaterial suspensions were dynamic, and nano-scale aggregates would settle into larger 1+ μm agglomerates within hours (see Additional file 1), so the μg/ml concentration is more accurate in the first few hours while the μg/cm^2^ concentration is more appropriate when the suspension has fully settled. Following a 24-h incubation with the nanomaterials, treatment medium was removed from the apical chamber to convert the inserts into air-liquid interface (ALI) cultures. Cultures were washed with ALI medium immediately following removal of the apical treatment and during replacement of basal chamber medium every 2 days until fixation/harvest at 28 days post-exposure. A fixed timepoint of 28 days was used in this study to allow for cultures from multiple donors to fully differentiate and control for variable rates of differentiation in each donor, though all had fully-differentiated before day 20. A post-differentiation treatment group was also examined, in which the untreated cultures were allowed to differentiate in ALI for 20 days and were then treated with 1 μg/cm^2^ MWNCTs for 24 h. These cells were fixed 7 days following treatment, at day 28 of differentiation.

### Lactate dehydrogenase assay

Cytotoxicity resulting from nanomaterial exposure was measured by lactate dehydrogenase (LDH) release into the apical chamber. Apical chambers were washed with phosphate buffered saline (PBS) 24 h prior to collection and accumulated LDH collected in a second apical wash was quantified using the CytoTox 98 colorimetric assay (Promega, Madison, WI). Absorbance at 495 nm by the conversion of the formazan dye product indicated the elevation of LDH concentrations and increased cytotoxicity. Total 24-h accumulated LDH release was normalized to vehicle control LDH (Additional file 2). While MWCNTs have been shown to interfere with colorimetric assays such as this [37], we have demonstrated in previous work [16] that the relatively low concentrations we utilize do not significantly alter assay results. Results of acellular assays containing only MWCNT or NG were subtracted from the treatment results to account for direct 495 nm absorbance by these materials, though these wells were not significantly different from media blanks (not shown).

### Whole mount immunocytochemistry

Culture inserts were fixed at day 28 following treatment and removal of apical medium (ALI day 28). Inserts were washed with PBS to remove mucus and fixed with 4% EM-grade paraformaldehyde (PFA) for 30 min. Cells were permeabilized with 0.2% TritonX-100 in PBS for 30 min, washed again with PBS, and blocked (with a solution of 1% BSA, 1% fish gelatin, 0.1% Triton-100, and 5% goat serum in PBS) for 1-h at room temperature. Primary incubation with rat anti-tubulin mAb, clone YL1/2 (EMD Millipore, Darmstadt, Germany, diluted to 5 μg/ml in blocking solution) was applied overnight at 4 °C. Wells were washed 3× for 30 min in 25% blocking solution in PBS before adding Alexa488-conjugated phalloidin (Thermo Fisher, Waltham, MA, 1:200) and Alexa647 anti-rat secondary antibody (Thermo Fisher, Waltham, MA, 1:200). Secondary incubation was performed for 2-h at room temperature, after which the membranes were washed with PBS 3× at 5 min each, excised from their plastic sprues, and mounted onto slides with Prolong gold plus DAPI (Thermo Fisher, Waltham, MA). Z-stacks taken from 10 μm of the apical surface of each membrane were imaged using a Zeiss 710 confocal microscope. Parameters such as gain, pinhole, laser intensity, z-planes per stack, and image post-processing were kept consistent between treatments. Eight z-stacks were taken from randomly-selected locations in the center of each membrane and used to produce maximum intensity projections. Pixel area analyses of these projections (using Image J software) were used to quantify ciliary tubulin staining and F-actin at tight junctions. Pixel areas were normalized to DAPI nuclear staining to account for reduced cellularity; however, cellularity was not altered by any treatment (see Additional file 4).

### Histological cross-section

ALI day 28 transwells were fixed with 4% PFA and dehydrated in plastic cassettes using 15 min submersions in ethanol (70%, 70, 90, 95, 100, 100%, and 100%) followed by three 15 min submersions in NeoClear xylene substitute (EMD Millipore, Darmstadt, Germany) prior to overnight paraffin embedding. Cross sections of differentiated membranes were stained with hematoxylin and eosin (H&E) and periodic acid-Schiff (PAS) to visualize cilia and mucins. Ciliated cells and goblet cells were counted using a 20× objective and results were expressed as average cell count per 500 μm length of membrane.

### Cilia beating analysis

Cultures at ALI day 28 were washed with ALI medium and 10 s of cilia beating was recorded with a Hamamatsu ORCA-Flash motion-capture camera using MetaMorph software (Molecular Devices, Sunnyvale, CA). Autoregressive spectral analysis was performed on fast Fourier transforms of image intensity which provided a spectrum of beating frequencies in each motion capture [38, 39]. The percentage of the total frequency spectrum which fell under the dominant (highest intensity) peak was calculated for each of 5 regions per membrane. These regions were selected based on the lack of free-floating debris during the 10 s recording and were otherwise selected “blindly” as beating differences could not be visually determined. Dominant amplitudes of cilia beating within regions of interest were also calculated by parametric reconstruction analysis. Analyses were performed using AutoSignal software, v1.7 (Systat Software, San Jose, CA).

### Quantitative PCR

RNA was collected from ALI day 1 and day 28 inserts and extracted using an RNeasy Plus Mini kit and columns by the manufacturer’s protocol (Qiagen, Venlo, Netherlands). Total RNA was converted to cDNA using the iScript reverse transcriptase kit (BioRad, Hercules, CA). QPCR was carried out using SYBR Green in an ABI (Waltham, MA) StepOne sequencer and primers for FOXJ1, MUC5AC, MUC5B, RARRES1, RDH12, and CRNN. 18S was used as an endogenous control and fold change in gene transcription was calculated by ddCt analysis. Primer sequences (QStar, Origene Technologies, Rockville, MD) can be found in Additional file 5.

### Ultrastructural imaging of cilia axonemes

Cell cultures were fixed in 3% glutaraldehyde at ALI day 28. Membranes were rinsed with PBS prior to post-fix in 1% osmium tetroxide. Membranes were then stained with uranyl acetate and dehydrated in the previously described ethanol series, followed by submersion in acetone. The samples were embedded in Polybed 812 epoxide resin. Membrane blocks were cut into thin sections of 80–90 nm, placed onto 200 mesh copper grids, and then stained again with uranyl acetate and lead citrate. Digital images were captured with a Orius SC1000/SC600 camera (Gatan, Pleasanton, CA) attached to a Tecnai T120 (FEI/Thermo Fisher, Wlatham, MA) transmission electron microscope (TEM). Images with clear, non-oblique, cross-sections of cilia were used to count abnormalities in axoneme 9 + 2 microtubule arrangement and/or dynein arm abnormalities. A total of 224 vehicle control-treated and 234 MWCNT-treated cilia cross-sections were counted for this purpose.

### Confocal microscopy of early ciliogenesis

Actin and γ-tubulin structure were examined in inserts fixed at ALI days 3, 5, and 14. Membranes were fixed with 4% EM-grade PFA for 15 min and excess aldehydes were quenched with 0.1 M glycine in PBS for 5 min. Cells were permeabilized with 0.1% TritonX-100 in PBS for 15 min and rinsed 3× with PBS. Membranes were blocked with 1% BSA and 5% goat serum in PBS for 1 h at room temperature. Rabbit anti-γ-tubulin mAb (Sigma-Aldrich, St. Louis, MO, diluted 1:800 in blocking solution) was applied overnight at 4 °C. Following three 5 min PBS washes, membranes were incubated with Alexa594-conjugated goat anti-rabbit and Alexa488-conjugated phalloidin (Thermo Fisher, Waltham, MA, diluted 1:1000 and 1:200 in 25% blocking solution, respectively) for 1-h at room temperature. Membranes were again washed with PBS (3× at 5 min each) before being excised from their plastic sprues and mounted onto slides with Prolong Gold. Z-stack images of the apical 10 μm of each membrane were taken with a Zeiss (Oberkochen, Germany) 880 confocal microscope using Airyscan imaging on Zen software (63× oil immersion objective). Parameters such as gain, pinhole, laser intensity, z-planes per stack, and image post-processing were kept consistent between treatments.

CEP164 and γ-tubulin were stained under similar conditions, using mouse anti-CEP164 (Sigma Aldrich, St. Louis, MO, diluted 1:800) and a blocking solution containing 10% goat serum and 5% BSA. Alexa488-conjugated goat anti-mouse and the previously mentioned Alexa594 anti-rabbit secondary antibodies (Thermo Fisher, Waltham, MA, diluted 1:1000 in blocking solution) were used to image CEP164 and γ-tubulin, respectively. Hoechst stain (diluted 1:1000) was used to identify cells (not shown in images). Isotype control primary antibodies were used to control for non-specific staining (found in Additional file 4). Confocal Z-stacks were taken of the apical 6 μm of each membrane, also using Airyscan imaging with a 4× software zoom (same microscope/objective).

### TEER measurement of differentiating ALI cultures

ALI cultures from one donor were used for TEER measurements during differentiation following nanomaterial treatment. Every 2 days after conversion to ALI, the apical chamber of each well was briefly filled with 300ul of ALI medium taken from the basal chamber (as to avoid introducing any new nutrients which would alter the cellular biochemistry during measurement) to permit TEER measurement. A single TEER measurement was recorded from each well using an ERS-2 Voltohmmeter (EMD Millipore, Darmstadt, Germany) with an STX01 probe, as per manufacturer’s instructions. Following each measurement, the apical medium was removed and fresh medium added to the basal chamber. Measurements were averaged from 3 wells for each treatment.

## Additional files

Additional file 1: Bronchoscopy procedure and the characterization of the nanomaterials. (DOCX 680 kb)

Additional file 2: Graph of cytotoxicity in ALI cultures, measured by LDH release, on days 1, 4, and 7 following MWCNT exposure. (DOCX 40 kb)

Additional file 3: ZO-1 tight junction staining in pre- vs post-differentiation exposed BECs. (DOCX 328 kb)

Additional file 4: Raw images of confocal Z-stacks used for Fig. 1, and images of isotype control antibodies used for Fig. 7 (DOCX 2950 kb)

Additional file 5: List of all primers and sequences used in the QPCR experiments (DOCX 13 kb)

## Abbreviations

ALI: Air-liquid interface
ANOVA: Analysis of variance
BECs: Bronchial epithelial cells
BSA: Bovine serum albumin
CoV: Control vehicle / dispersion medium alone
DPPC: 1,2-dipalmitoyl-sn-glycero-3-phosphocholine
H&E: Hematoxylin and eosin
LDH: Lactate dehydrogenase
MWCNTs: Multi-walled carbon nanotubes
NG: Mesoporous nanoscale graphitized carbon
PAS: Periodic Acid-Schiff
PBS: Phosphate buffered saline
TEER: Trans-epithelial electrical resistance
TEM: Transmission electron microscopy
