# Supplementary material for: Impaired Ciliogenesis in differentiating human bronchial epithelia exposed to non-Cytotoxic doses of multi-walled carbon Nanotubes
Source: Part Fibre Toxicol. 2017 Nov 13;14:44. doi: 10.1186/s12989-017-0225-1 (PMC5683528; doi:10.1186/s12989-017-0225-1)
Supplement: Supplementary file 1 — Bronchoscopy procedure and the characterization of the nanomaterials. (DOCX 680 kb) [file 12989_2017_225_MOESM1_ESM.docx]

**Impaired Ciliogenesis in Differentiating Human Bronchial Epithelia Exposed to Non-Cytotoxic Doses of Multi-Walled Carbon Nanotubes**

**Additional File 1**

***Ryan J. Snyder,*** *^†^****^*^ Salik Hussain,****^†^* ***Charles J. Tucker,*** *^†^*

***Scott H. Randell,*** *^‡^* ***and Stavros Garantziotis****^†^*

^†^ National Institute of Environmental Health Sciences (NIEHS)/National Institute of Health (NIH), Research Triangle Park 27709, NC, USA

^‡^University of North Carolina Chapel Hill, Chapel Hill 27599-7248, NC, United States

*** Corresponding Author**

Ryan J. Snyder

Clinical Research Unit,

National Institute of Environmental Health Sciences,

Research Triangle Park,

27709, Durham, NC.

Tel: +1 919 316 4836

Fax: +1 919 541 9854

E-mail: [snyder3@niehs.nih.gov](mailto:snyder3@niehs.nih.gov)

*Collection of Bronchial Epithelial Cells*

Bronchoscopy of healthy human donors and the use of cells derived from this procedure was performed after review and approval by the National Institute of Environmental Health Sciences Intramural Review Board (NIEHS-IRB, clinicaltrials.gov identifier: NCT01224691). Bronchoscopies were performed with the donors’ written consent by a trained pulmonologist at the NIEHS Clinical Research Unit (CRU). Bronchoscopies proceeded per the methods described in NIH protocol 11-E-0006. Described briefly, a 270 mL volume of sterile saline, 1 aliquot of 20 mL, and 5 additional aliquots of 50 mL each, were injected/aspirated through the bronchoscope channel by syringe. The lavage fluids were added to polypropylene tubes and kept on ice. A total of up to 3 to 5 cytology brushings were conducted per donor to obtain 4 to 6 million surface mucosal epithelial cells. After each brushing, the brush was removed and the recovered cells were dislodged from the brush by stirring in a 15mL conical vial containing Lonza BEGM medium (supplemented with 2x amphotericin B). Undifferentiated epithelial cells were selected by growth in the serum-free medium.

*Nanomaterial Characterization*

MWCNTs used in this study have been extensively characterized by Ryman-Rasmussen, et al (*Am J Resp Cell Mol,* 2009), who generously donated the particles used in this study. Their analysis revealed >94% purity by thermogravimetric analysis (TGA). Impurities identified by IC-AES (inductively coupled plasma Auger electron spectroscopy) were primarily oxygen (0.63%) and nickel (0.34%), with trace amounts of lanthanum (0.03%). Average length and diameter determined by TEM were 0.3-50µm and 30-50nm respectively. Surface area, determined by Brunauer-Emmett-Teller (BET) method, was found to be 109.29 m^2^/g. Measurements were mostly consistent with those of the manufacturer (Helix Nanomaterial Solutions). The control material (graphitized mesoporous nanocarbon, NG) was characterized by the manufacturer (Sigma Aldrich). Purity was determined to be >99.95%, with only trace metals present, and particle size determined to be <500nm in diameter. Surface area was measured at 70m^2^/g with a 137Å average pore diameter. Dynamic light scattering measurements performed in our laboratory showed similar hydrodynamic diameters between MWCNT and NG agglomerates 1hr and 24hrs following sonication/dispersion (see figure below). Note that much larger percentages of 1µm+ agglomerates are found after 24hrs in suspension, and that the derived count rate dropped during this time from 21539 kcps to 12612 kcps, suggesting significant settling over the 24 hour treatment period.


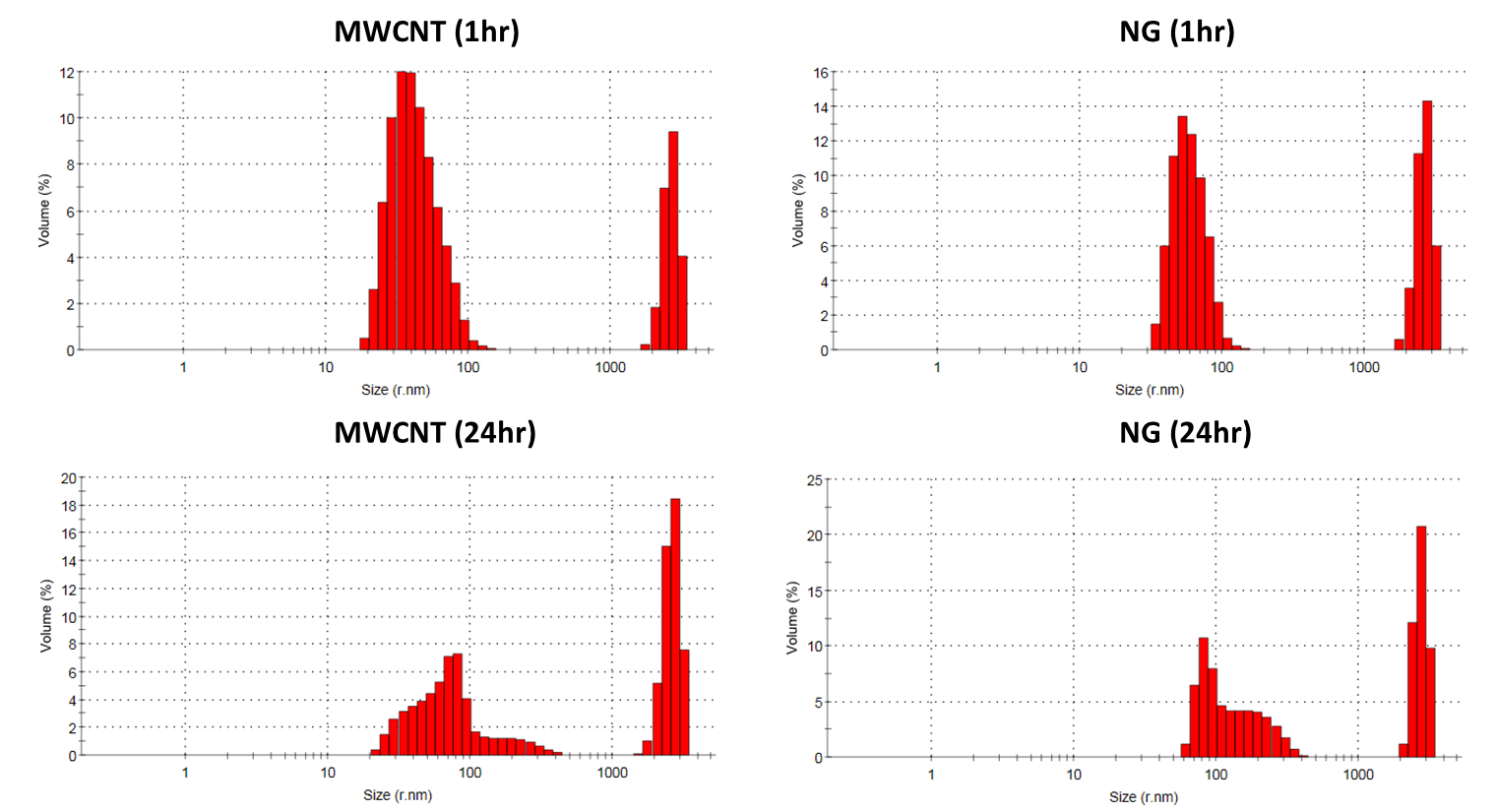


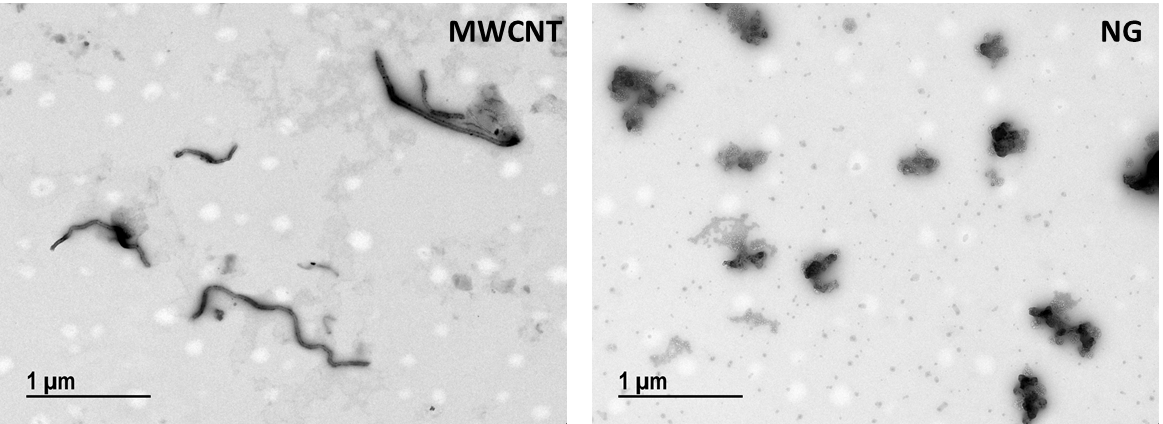
Electron micrographs of the MWCNT fibers and nanographite (NG) particles following sonication are provided below. Fibers are of variable length and relatively disaggregated prior to treatments.
