# Supplementary material for: Impaired Ciliogenesis in differentiating human bronchial epithelia exposed to non-Cytotoxic doses of multi-walled carbon Nanotubes
Source: Part Fibre Toxicol. 2017 Nov 13;14:44. doi: 10.1186/s12989-017-0225-1 (PMC5683528; doi:10.1186/s12989-017-0225-1)
Supplement: Supplementary file 2 — Graph of cytotoxicity in ALI cultures, measured by LDH release, on days 1, 4, and 7 following MWCNT exposure. (DOCX 40 kb) [file 12989_2017_225_MOESM2_ESM.docx]

**Impaired Ciliogenesis in Differentiating Human Bronchial Epithelia Exposed to Non-Cytotoxic Doses of Multi-Walled Carbon Nanotubes**

**Additional File 2**

***Ryan J. Snyder,*** *^†^****^*^ Salik Hussain,****^†^* ***Charles J. Tucker,*** *^†^*

***Scott H. Randell,*** *^‡^* ***and Stavros Garantziotis****^†^*

^†^ National Institute of Environmental Health Sciences (NIEHS)/National Institute of Health (NIH), Research Triangle Park 27709, NC, USA

^‡^University of North Carolina Chapel Hill, Chapel Hill 27599-7248, NC, United States

*** Corresponding Author**

Ryan J. Snyder

Clinical Research Unit,

National Institute of Environmental Health Sciences,

Research Triangle Park,

27709, Durham, NC.

Tel: +1 919 316 4836

Fax: +1 919 541 9854

E-mail: [snyder3@niehs.nih.gov](mailto:snyder3@niehs.nih.gov)


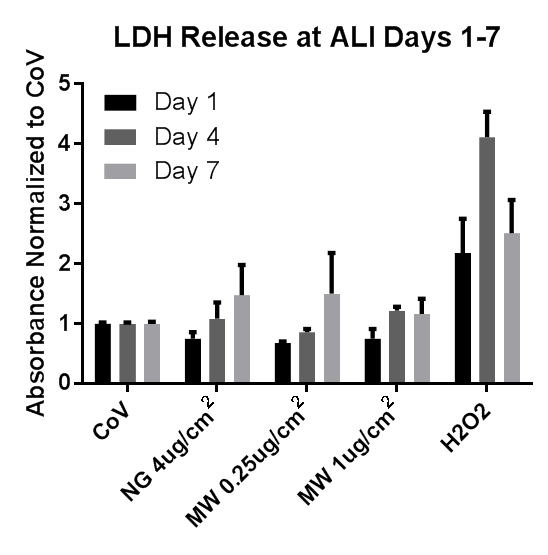


**Cytotoxicity measured by LDH release:** LDH release into apical chamber over a 24 hour period, at ALI days 1, 4, and 7. Values are expressed as fold increase over dispersion vehicle control absorbance at 495nm wavelength (N=3, bars indicate SD). While MWCNTs are known to interfere with colorimetric assays, acellular testing found that these lower doses did not interfere with the LDH assay. Cytotoxicity of MWCNTs in ALI culture was found to be negligible in doses 1ug/cm^2^ or lower.

*Lactate Dehydrogenase Assay*

Cytotoxicity resulting from nanomaterial exposure was measured by lactate dehydrogenase (LDH) release into the apical chamber. Apical chambers were washed with phosphate buffered saline (PBS) 24 hours prior to collection and accumulated LDH collected in a second apical wash was quantified using the CytoTox 98 colorimetric assay (Promega). Absorbance at 495nm by the conversion of the formazan dye product indicated the elevation of LDH concentrations and increased cytotoxicity. Total 24 hour accumulated LDH release was normalized to vehicle control LDH. While MWCNTs have been shown to interfere with colorimetric assays such as this^23^, we have demonstrated in previous work^16^ that the relatively low concentrations we utilize do not significantly alter assay results. Results of acellular assays containing only MWCNT or NG were subtracted from the treatment results to account for direct 495nm absorbance by these materials, though these wells were not significantly different from media blanks (not shown).
