# Supplementary material for: Impaired Ciliogenesis in differentiating human bronchial epithelia exposed to non-Cytotoxic doses of multi-walled carbon Nanotubes
Source: Part Fibre Toxicol. 2017 Nov 13;14:44. doi: 10.1186/s12989-017-0225-1 (PMC5683528; doi:10.1186/s12989-017-0225-1)
Supplement: Supplementary file 3 — ZO-1 tight junction staining in pre- vs post-differentiation exposed BECs. (DOCX 328 kb) [file 12989_2017_225_MOESM3_ESM.docx]

**Impaired Ciliogenesis in Differentiating Human Bronchial Epithelia Exposed to Non-Cytotoxic Doses of Multi-Walled Carbon Nanotubes**

**Additional File 3**

***Ryan J. Snyder,*** *^†^****^*^ Salik Hussain,****^†^* ***Charles J. Tucker,*** *^†^*

***Scott H. Randell,*** *^‡^* ***and Stavros Garantziotis****^†^*

^†^ National Institute of Environmental Health Sciences (NIEHS)/National Institute of Health (NIH), Research Triangle Park 27709, NC, USA

^‡^University of North Carolina Chapel Hill, Chapel Hill 27599-7248, NC, United States

*** Corresponding Author**

Ryan J. Snyder

Clinical Research Unit,

National Institute of Environmental Health Sciences,

Research Triangle Park,

27709, Durham, NC.

Tel: +1 919 316 4836

Fax: +1 919 541 9854

E-mail: [snyder3@niehs.nih.gov](mailto:snyder3@niehs.nih.gov)

*Primer Sequences Used for QPCR*

QStar primers were used at manufacturer recommended concentrations (Origene Technologies, Rockville, MD) and their sequences are as follows (5’->3’):

FOXJ1 (Forward)- ACTCGTATGCCACGCTCATCTG

FOXJ1 (Reverse)- GAGACAGGTTGTGGCGGATTGA

MUC5AC (Forward)- CCACTGGTTCTATGGCAACACC

MUC5AC (Reverse)- GCCGAAGTCCAGGCTGTGCG

MUC5B (Forward)- CTGCTACGACAAGGACGGAAAC

MUC5B (Reverse)- AAGGCTGTGAGCGCACTGGATG

RARRES1 (Forward)- TCACGTGGTCTTCAGCACAGAG

RARRES1 (Reverse)- TTTCTCGATGAGCCGTGTACAAG

RDH12 (Forward)- TCTATCCGAGCCTTTGCTGAGG

RDH12 (Reverse)- GGTGGGTTTCAAAGCCATCAGC

CRNN (Forward)- GGAGCTGAAAAGACTCTTGGAGC

CRNN (Reverse)- CTGTGTGGTCTTCATCCAGCAG

18S (Forward)- GTAACCCGTTGAACCCCATT

18S (Reverse)- CCATCCAATCGGTAGTAGCG
