# Supplementary material for: Impaired Ciliogenesis in differentiating human bronchial epithelia exposed to non-Cytotoxic doses of multi-walled carbon Nanotubes
Source: Part Fibre Toxicol. 2017 Nov 13;14:44. doi: 10.1186/s12989-017-0225-1 (PMC5683528; doi:10.1186/s12989-017-0225-1)
Supplement: Supplementary file 4 — Raw images of confocal Z-stacks used for Fig. 1, and images of isotype control antibodies used for Fig. 7 (DOCX 2950 kb) [file 12989_2017_225_MOESM4_ESM.docx]

**Impaired Ciliogenesis in Differentiating Human Bronchial Epithelia Exposed to Non-Cytotoxic Doses of Multi-Walled Carbon Nanotubes**

**Additional File 4**

***Ryan J. Snyder,*** *^†^****^*^ Salik Hussain,****^†^* ***Charles J. Tucker,*** *^†^*

***Scott H. Randell,*** *^‡^* ***and Stavros Garantziotis****^†^*

^†^ National Institute of Environmental Health Sciences (NIEHS)/National Institute of Health (NIH), Research Triangle Park 27709, NC, USA

^‡^University of North Carolina Chapel Hill, Chapel Hill 27599-7248, NC, United States

*** Corresponding Author**

Ryan J. Snyder

Clinical Research Unit,

National Institute of Environmental Health Sciences,

Research Triangle Park,

27709, Durham, NC.

Tel: +1 919 316 4836

Fax: +1 919 541 9854

E-mail: [snyder3@niehs.nih.gov](mailto:snyder3@niehs.nih.gov)


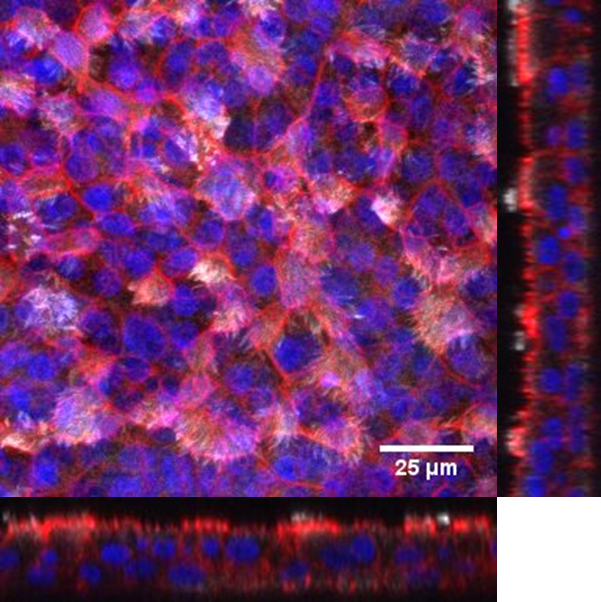

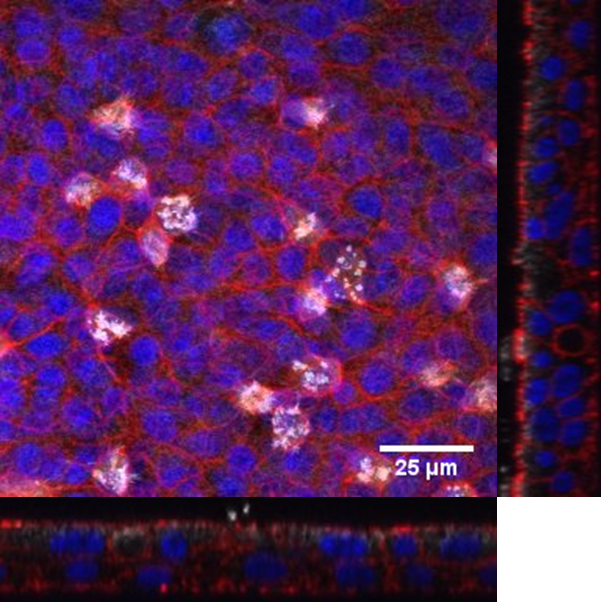

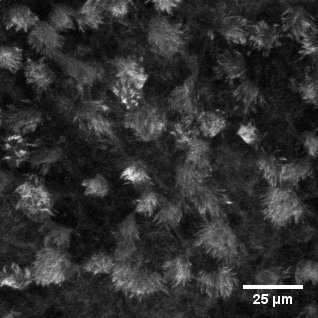

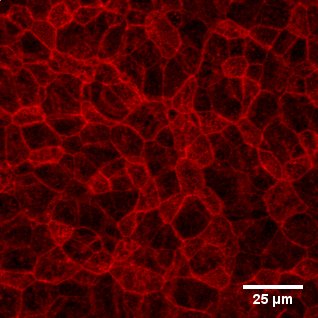

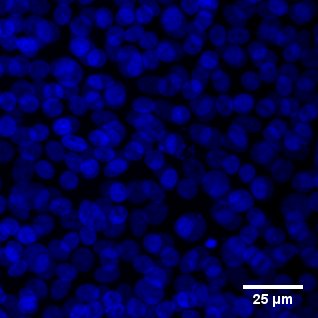

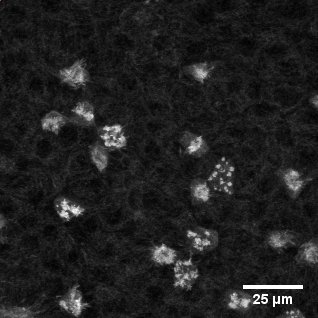

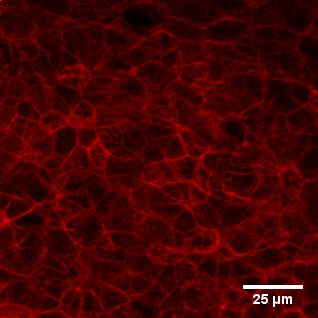

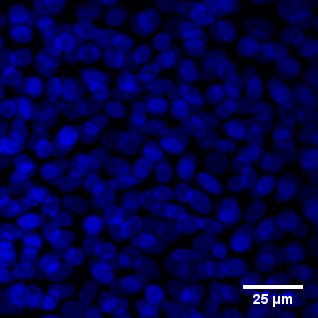


**Control Vehicle- Composite**

**MWCNT 1µg/cm^2^- Composite**

**Raw Images from Figure 1.** Examples of confocal Z-stacks taken prior to threshold processing and pixel area analysis for Figure 1. Top images are composite summation images from ALI day 28 cultures treated prior to differentiation with control dispersion vehicle and MWCNT 1µg/cm^2^, as well as orthogonal views showing the Z-plane staining distribution. Below these images are α-tubulin, F-actin, and nuclear DAPI staining, respectively. Tubulin and actin staining are both visibly attenuated with MWCNT exposure. DAPI nuclear staining remains consistent between treatments, indicating no loss of cellularity despite the reductions in cilia and F-actin.

**MWCNT 1µg/cm^2^- Tubulin**

**Control Vehicle- Tubulin**

**MWCNT 1µg/cm^2^- Actin**

**Control Vehicle- Actin**

**MWCNT 1µg/cm^2^**

**Control Vehicle**

**MWCNT 1µg/cm^2^- DAPI**

**Control Vehicle- DAPI**

**B)**

**A)**

Composite

Rabbit IgG + Alexa594 Anti-rabbit

Anti-CEP164 + Alexa488 Anti-mouse

Composite

Anti-tubulin + Alexa594 Anti-rabbit

Mouse IgG2 + Alexa488 Anti-mouse

**Isotype controls for Figure 7.** Isotype control staining to verify that anti-CEP164 and anti-y-tubulin antibodies in Figure 7 were not binding non-specifically. A) In the original study, CEP164 was stained with a mouse IgG2 primary, so here a mouse IgG2 non-specific isotype primary was used for this experiment in its place, along with the same anti-mouse Alexa488-conjugated secondary. B) Likewise, y-tubulin was stained with a rabbit IgG primary, and was replaced in this study with a rabbit IgG non-specific isotype primary, and the same anti-rabbit Alexa594-conjugated secondary. No non-specific staining and very few staining artifacts appeared in the isotype control images, indicating that the primary antibodies were specific to their targets and that the wash steps were successful.
