# Supplementary material for: Impaired Ciliogenesis in differentiating human bronchial epithelia exposed to non-Cytotoxic doses of multi-walled carbon Nanotubes
Source: Part Fibre Toxicol. 2017 Nov 13;14:44. doi: 10.1186/s12989-017-0225-1 (PMC5683528; doi:10.1186/s12989-017-0225-1)
Supplement: Supplementary file 5 — List of all primers and sequences used in the QPCR experiments (DOCX 13 kb) [file 12989_2017_225_MOESM5_ESM.docx]

**Impaired Ciliogenesis in Differentiating Human Bronchial Epithelia Exposed to Non-Cytotoxic Doses of Multi-Walled Carbon Nanotubes**

**Additional File 5**

***Ryan J. Snyder,*** *^†^****^*^ Salik Hussain,****^†^* ***Charles J. Tucker,*** *^†^*

***Scott H. Randell,*** *^‡^* ***and Stavros Garantziotis****^†^*

^†^ National Institute of Environmental Health Sciences (NIEHS)/National Institute of Health (NIH), Research Triangle Park 27709, NC, USA

^‡^University of North Carolina Chapel Hill, Chapel Hill 27599-7248, NC, United States

*** Corresponding Author**

Ryan J. Snyder

Clinical Research Unit,

National Institute of Environmental Health Sciences,

Research Triangle Park,

27709, Durham, NC.

Tel: +1 919 316 4836

Fax: +1 919 541 9854

E-mail: [snyder3@niehs.nih.gov](mailto:snyder3@niehs.nih.gov)


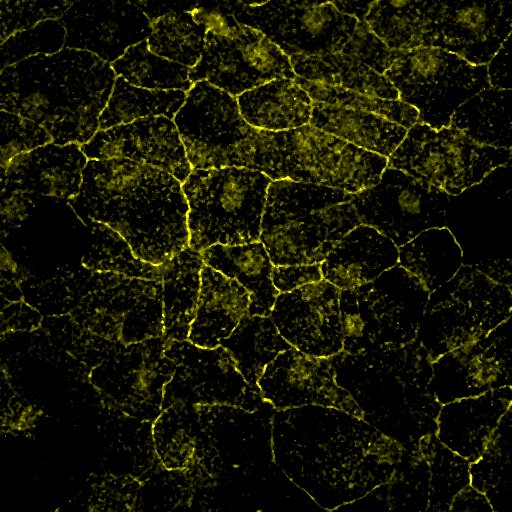

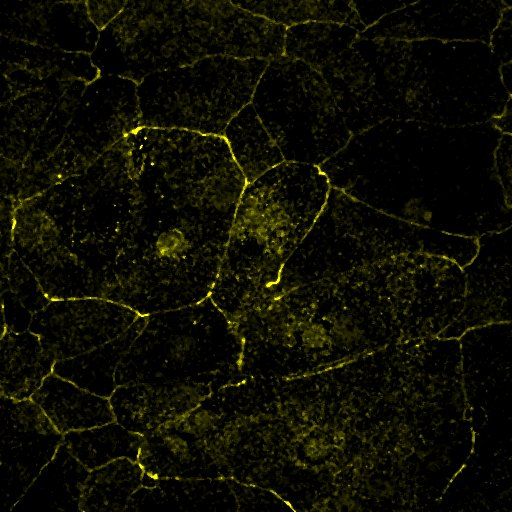

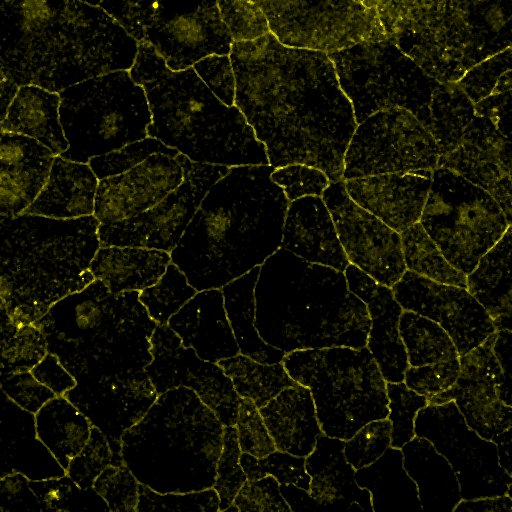




**Exposure at Day 1 (Pre)**

**Exposure at Day 20 (Post)**

**A)**

**Controls**

**MWCNT**

**1µg/cm^2^**

**B)**

**ZO-1 staining.** A) Staining for ZO-1 at the tight junctions was performed in cultures treated at days 1 (“Pre”) and 20 (“Post”) with MWCNTs or media alone. Experiments were performed using a single donor with 3 replicate wells. Membranes were fixed at ALI-day 28 in 4% PFA, blocked with 10% goat serum, and stained with anti-ZO-1 mAb (Santa Cruz) and Hoechst. B) Pixel density analysis of ZO-1 staining normalized to nuclei count. No statistically-significant differences in ZO-1 staining were found between any treatments. A noticeable decrease in ZO-1 staining can be observed in the pre-differentiation cultures treated with MWCNT 1µg/cm^2^, though this is due to a substantial increase in cell size, rather than reduced tight junction staining.
